# Improving human mesenchymal stem cell-derived hepatic cell energy metabolism by manipulating glucose homeostasis and glucocorticoid signaling

**DOI:** 10.3389/fendo.2022.1043543

**Published:** 2023-01-13

**Authors:** Joana Saraiva Rodrigues, Andreia Faria-Pereira, Sérgio Póvoas Camões, Ana Sofia Serras, Vanessa Alexandra Morais, Jorge Lira Ruas, Joana Paiva Miranda

**Affiliations:** ^1^ Research Institute for Medicines (imed.ULisboa), Faculty of Pharmacy, Universidade de Lisboa, Lisbon, Portugal; ^2^ Instituto de Medicina Molecular João Lobo Antunes, Faculdade de Medicina, Universidade de Lisboa, Lisbon, Portugal; ^3^ Department of Physiology and Pharmacology, Biomedicum, Karolinska Institutet, Stockholm, Sweden

**Keywords:** alternative hepatic *in vitro* models, mesenchymal stem cells, hepatocyte-like cells, insulin, glucose, dexamethasone, metabolism

## Abstract

**Introduction:**

The development of reliable hepatic *in vitro* models may provide insights into disease mechanisms, linking hepatocyte dysmetabolism and related pathologies. However, several of the existing models depend on using high concentrations of hepatocyte differentiation-promoting compounds, namely glucose, insulin, and dexamethasone, which is among the reasons that have hampered their use for modeling metabolism-related diseases. This work focused on modulating glucose homeostasis and glucocorticoid concentration to improve the suitability of a mesenchymal stem-cell (MSC)-derived hepatocyte-like cell (HLC) human model for studying hepatic insulin action and disease modeling.

**Methods:**

We have investigated the role of insulin, glucose and dexamethasone on mitochondrial function, insulin signaling and carbohydrate metabolism, namely AKT phosphorylation, glycogen storage ability, glycolysis and gluconeogenesis, as well as fatty acid oxidation and bile acid metabolism gene expression in HLCs. In addition, we evaluated cell morphological features, albumin and urea production, the presence of hepatic-specific markers, biotransformation ability and mitochondrial function.

**Results:**

Using glucose, insulin and dexamethasone levels close to physiological concentrations improved insulin responsiveness in HLCs, as demonstrated by AKT phosphorylation, upregulation of glycolysis and downregulation of *Irs2* and gluconeogenesis and fatty acid oxidation pathways. Ammonia detoxification, EROD and UGT activities and sensitivity to paracetamol cytotoxicity were also enhanced under more physiologically relevant conditions.

**Conclusion:**

HLCs kept under reduced concentrations of glucose, insulin and dexamethasone presented an improved hepatic phenotype and insulin sensitivity demonstrating superior potential as an *in vitro* platform for modeling energy metabolism-related disorders, namely for the investigation of the insulin signaling pathway.

## Introduction

Obesity, considered a global epidemic and a major cause of death, is closely related to insulin resistance (IR) that may lead to non-alcoholic fatty liver disease (NAFLD) and type 2 diabetes (T2D). Playing a key role in the control of systemic glucose and lipid metabolism, the liver is a crucial insulin target organ. It is the main source of endogenous glucose, produced *via* gluconeogenesis, and thus has a key part in IR and associated disorders. Moreover, insulin has a direct action on the control of glucose metabolism in hepatocytes (1). Therefore, identifying the specific role of insulin on hepatocyte metabolism in physiological and in IR conditions is crucial to unravel the link between IR and related pathological conditions. However, for this field to progress there is a need for human-based physiologically relevant hepatic culture models that enable investigating insulin action in the hepatocyte and its role in metabolic homeostasis and disease progression.

The well-described drawbacks associated to the use of human primary hepatocytes (hpHeps) have promoted a growing interest within the scientific community for developing reliable human physiological hepatic models, namely by deriving hepatocyte-like cells (HLCs) from induced pluripotent stem cells (iPSCs), mesenchymal stem cells (MSCs) or other stem cells (2–6). In particular, our group has previously characterized and validated the use of MSC-derived HLCs, namely in comparison with other cell lines, which support the use of these HLCs for other applications, namely as an *in vitro* platform for disease modeling (4, 5). Still, a fully mature and stable hepatic phenotype has not been reached yet. Strategies to counteract the loss of hepatocyte function, viability and the differentiated phenotype in culture rely deeply on the use of differentiation-promoting compounds, such as glucose, dexamethasone and insulin (7–11).

Although it is appreciated that dexamethasone and other glucocorticoids can differentially regulate some drug-detoxifying enzymes, such as cytochrome P450 (CYP) enzymes, it is not clear what their role is in hepatic differentiation. In fact, dexamethasone selectively induces or inhibits some CYP isoforms while hyper- and hypo-glycemic conditions also cause differences in the functionality of different CYP isoforms (12–14). Moreover, the involvement of glucose or insulin in hepatic differentiation is also not fully understood, and the concentrations used are often far from human physiological values (within the millimolar range for glucose and nanomolar range for insulin). This ultimately interferes with energy homeostasis and eventually causes IR *in vitro*, compromising the use of these models for studying metabolic disease development or progression (13). As for the insulin signaling pathway in *in vitro* hepatic models, this remains largely uncharacterized. A recent study has directly compared insulin action in several hepatic cell lines, showing that none of them resemble pHeps in terms of energy metabolism (1). The authors attributed the inadequacy of the different cell lines for metabolism studies or disease modeling to defective levels of gluconeogenic enzymes and glucose production or insulin unresponsiveness.

Here, we investigated the effects of reducing insulin and dexamethasone levels in glucose metabolism, biotransformation ability, mitochondrial activity, as well as ammonia detoxification and albumin secretion. We further analyzed the expression of genes involved in glycolysis, gluconeogenesis, fatty acid (FA) and bile acid metabolism, and mitochondrial function and if it correlates with an improvement in the metabolic profile of cells.

## Materials and methods

All culture media and supplements, solvents (all analytical grade) and other chemicals were acquired from Sigma-Aldrich (Madrid, Spain) unless specified.

### Cell culture

The isolation of human neonatal mesenchymal stem cells (hnMSCs) was approved by the Ethics Committee of the Cascais Hospital Dr. José de Almeida. hnMSCs isolated from human umbilical cord stroma were fully characterized (15) and cultured as described by Santos et al. (16). For generating HLCs, a three-step differentiation protocol was applied to hnMSCs as detailed previously (4). From D21 onwards, HLCs seeded in collagen-coated culture plates were maintained in one of four media formulations comprehending different glucose, insulin, and dexamethasone concentrations supplemented with 1% of DMSO, 8 ng/mL of oncostatin M (OSM), 1% of penicillin-streptomycin (PS) and 0.01% of amphotericin B (Ampho): (i) Diff, as detailed previously (4), with Iscove’s modified Dulbecco’s medium (IMDM) containing 25 mM of glucose, 1.72 µM of insulin and 1 µM of dexamethasone; (ii) Diff ^-glu^, with Dulbecco’s modified Eagle’s medium (DMEM), supplemented with L-glutamic acid, L-proline, HEPES and alanine to match IMDM concentrations, containing 5 mM of glucose, 1.72 µM of insulin and 1 µM of dexamethasone; (iii) Physiol ^+glu^, with IMDM supplemented with 1 nM of insulin, 100 nM of dexamethasone and 0.2% of bovine serum albumin (BSA); and (iv) Physiol, with DMEM supplemented with 1 nM of insulin, 100 nM of dexamethasone and 0.2% BSA (**Table 1**).

**Table 1:** Concentrations of glucose, insulin and dexamethasone present in Diff and Physiol media.

	Diff	Physiol
Concentrations	25 mM of glucose 1.72 µM of insulin 1 µM of dexamethasone	5 mM of glucose 1 nM of insulin 100 nM of dexamethasone

HepG2 cells (ATCC, MD, USA) and cryopreserved hpHeps (pool of 10 donors; Invitrogen, CA, USA) were cultured as described previously (4, 5).

### Collagen coating

The protocol for rat-tail extraction was performed as described by Rajan et al. (17). The extracted rat-tail collagen was dissolved in 0.1% acetic acid to a stock concentration of 1 mg/mL. The stock solution was diluted in PBS to 0.2 mg/mL in a volume that assures total culture surface coverage. After 1h-incubation at 37 °C, cell culture surfaces were washed with PBS before inoculation. The differentiation process occurred using this collagen coating until D17 in T-flasks and onwards for cultures in well plates.

### Urea and albumin production

Urea and albumin were quantified in cell culture supernatants using a colorimetric urea kit (QuantiChrom^™^ Urea Assay Kit, BioAssay Systems, Hayward, CA, USA) and an enzyme-linked immunosorbent assay (ELISA) kit (Bethyl Laboratories, Montgomery, TX, USA), respectively. The absorbance was measured at 520 nm for urea and 450 nm for albumin in a microplate reader (SPECTROStar Omega, BMG Labtech, Stuttgart, Germany), according to manufacturer’s instructions. Data is presented as the rate of production: µg/10^6^ cells.h (for urea) and pg/10^6^ cells.h (for albumin).

### Biotransformation activity

EROD assay covers CYP1A1 and CYP1A2 activity (12, 18–25). The protocol herein used was adapted from Donato et al. (26) and consisted in a 90-minute cell incubation with 8 µM of 7-ethoxyresorufin followed by a 2-hour enzymatic digestion with β-glucuronidase/arylsulfatase. The concentration of the product (7-hydroxyresorufin) was measured at an excitation wavelength of 530 nm and an emission of 590 nm.

UGTs’ activity was determined by quantification of 4-methylumbelliferone (4-MU) before and after cell incubation to evaluate the extent of substrate conversion, as described by Miranda et al. (27, 28).

Protein quantification was performed as detailed in Cipriano et al. (4). EROD and UGT activities were normalized to incubation time (h) and cell number (10^6^ cells).

### Mitochondrial function

HLC mitochondrial function was assessed by direct measurement of the oxygen consumption rate (OCR) using extracellular flux analysis (XF24, Seahorse Biosciences, North Billerica, MA, USA). HLCs were inoculated in pre-coated Seahorse XF cell culture plates as described in section Cell culture’ methods section. The Mitochondrial Stress Test assay was performed in XF Base medium (Agilent Technologies, Santa Clara, CA, USA) with 1 mM of pyruvate, 2 mM of L-glutamine (ThermoFisher Scientific, Waltham, MA, USA) and 10 mM of glucose. Baseline OCR were measured every 7 minutes. Following baseline measurements, oligomycin (1.5 µM), Carbonyl cyanide-4-(trifluoromethoxy)phenylhydrazone (FCCP) (1.25 µM), and rotenone (2 µM) and antimycin A (2 µM) were sequentially injected to measure OCR. From the obtained OCR profile, basal respiration, ATP production, maximal respiration and spare respiratory capacity could be calculated. Protein extraction from the HLCs was performed with RIPA lysis buffer supplemented with protease inhibitors 1x (Roche, Basel, Switzerland). Protein concentration (µg/µL) was determined using the bicinchoninic acid (BCA) protein assay kit (Pierce) according to manufacturer instructions. Mitochondrial function was normalized to the protein concentration (µg/µL) of each well.

### HLC viability assessment upon paracetamol exposure

Paracetamol cytotoxicity was evaluated using the MTS reduction assay (Promega, Madison, WI, USA). At D34, cells were exposed to concentrations of paracetamol of 0, 5, 10, 15, 20, 25, 50 and 75 mM. Cell viability was measured upon a 24-hour incubation, according to manufacturer’s instructions. IC_50_ was calculated with a nonlinear regression fit for the Log_10_ transformation of the concentration values using GraphPad Prism (GraphPad Software, La Jolla, CA, USA). The percentage of viable cells was calculated relative to non-treated HLCs.

### Periodic acid Schiff’s staining

PAS staining was performed as described previously (4, 5). The wells were rinsed with distilled water and were observed under light microscope (Olympus CK30 inverted microscope, Tokyo, Japan).

### Insulin stimuli

The hormone stimuli assays were performed at D34, exposing cells to 80 nM of insulin for 8 hours for gene expression analysis and for 30 minutes for AKT phosphorylation analysis. Before the insulin stimuli, cells culture medium was changed to starvation medium (DMEM, 1% of PS, 4 mM of glutamine, 1% of DMSO, 8 ng/mL of OSM and 0.2% of BSA), for an incubation period of 2 hours, to enhance the response to insulin incubation, as described in Correia et al. (29).

### Gene expression

Total RNA of 6.0×10^5^ cells was isolated using TRIzol (Life Technologies, Carlsbad, CA, USA) and extracted according to the manufacturer’s instructions. RNA concentration was determined by measuring the absorbance at 260 nm using LVis plate mode (SPECTROstar Omega, BMG Labtech). cDNA was synthesized from 1 μg of RNA using NZY First-Strand cDNA Synthesis Kit (NZYTech, Lisbon, Portugal), according to the manufacturer’s instructions. Quantitative real-time polymerase chain reaction (RT-qPCR) was performed using PowerUp SYBR Green Master Mix (Life Technologies) for a final reaction volume of 15 μL, using 2 μL of template cDNA and 0.333 μM of forward and reverse primers. Primer sequences are provided in **Table 2**. Reaction was performed on QuantStudio^™^ 7 Flex Real-Time PCR System (Applied Biosystems, Foster City, CA, USA) according to the described by Cipriano et al. (4, 5). The comparative Ct method (2-^ΔΔCt^) was used to quantify gene expression, which was normalized to a reference gene (β-actin).

**Table 2:** Primers used for RT-qPCR characterization of HLCs, undifferentiated hnMSCs and hpHeps.

Gene	Forward sequence (5’-3’)	Reverse sequence (5’-3’)
*β-actin*	CATGTACGTTGCTATCCAGGC	CTCCTTAATGTCACGCACGAT
*Ck-19*	ATGGCCGAGCAGAACCGGAA	CCATGAGCCGCTGGTACTCC
*Cyp3a4*	ATTCAGCAAGAAGAACAAGGACA	TGGTGTTCTCAGGCACAGAT
*Hnf-4a*	ATTGACAACCTGTTGCAGGA	CGTTGGTTCCCATATGTTCC
*Alb*	TGCTTGAATGTGCTGATGACAGGG	AAGGCAAGTCAGCAGGCATCTCATC
*Ck-18*	TGGTACTCTCCTCAATCTGCTG	CTCTGGATTGACTGTGGAAGT
*Pdk4*	TCTGAGGCTGATGACTGGTG	GGAGGAAACAAGGGTTCACA
*Pepck*	GCTTTTCAGCATCTCCAAGGA	GCTTCAAGGCAAGGATCTCTC
*G6pase*	CAGAGCAATCACCACCAAGC	ACATTCATTCCTTCCTCCATCC
*Ppara*	CTGTCATTCAAGCCCATCTTC	TTATTTGCCACAACCCTTCC
*Cpt1a*	TCCAGTTGGCTTATCGTGGTG	TCCAGAGTCCGATTGATTTTTGC
*Acox1*	ACTCGCAGCCAGCGTTATG	AGGGTCAGCGATGCCAAAC
*Fxr*	AGAACCTGGAAGTGGAACC	CTCTGCTACCTCAGTTTCTCC
*Ppargc1a*	GCTGAAGAGGCAAGAGACAGA	AAGCACACACACCACACACA
*Irs2*	CGGTGAGTTCTACGGGTACAT	TCAGGGTGTATTCATCCAGCG

### Western blot analysis

Western blot analysis was performed on HLC lysates. 30 μg of protein, quantified by Bradford protein assay kit according to manufacturer instructions, were separated by 12% SDS-PAGE and were transferred to PVDF membrane. Rabbit monoclonal antibody against human AKT (1:1000; Cell Signaling Technology, Danvers, MA, USA) and rabbit monoclonal antibody against human p-AKT (1:1000; Cell Signaling Technology) were used as primary antibodies. All blots were probed overnight at 4°C. Anti-rabbit horseradish peroxidase-conjugated antibody was used as secondary (1:20 000; Jackson ImmunoResearch, Ely, Cambridgeshire, UK). Immunoreacted proteins were detected by using Immobilon Western Blotting Kit (Merck Millipore, Burlington, MA, USA).

### Statistical analysis

The results are presented as Average ± SEM unless stated otherwise. Data was analyzed with two-way ANOVA with GraphPad Prism. A threshold of *p* < 0.05 was considered statistically significant.

## Results

### Reducing glucose, insulin and dexamethasone concentrations enhances the hepatic phenotype and biotransformation competence of HLCs

Ideally*, in vitro* hepatic models should retain most, if not all, of the characteristic biochemical machinery and molecular pathways that allow for a normal phenotype. We evaluated the effect of glucose, insulin and dexamethasone levels on HLC maturation by analyzing hepatic morphological features, albumin and urea production, as well as the presence of hepatic-specific markers and the biotransformation ability of HLCs at day 27 (D27) and day 34 (D34), corresponding to 1 and 2 weeks in culture post differentiation, respectively. As such, a comparative analysis of glucose, insulin and dexamethasone at physiological levels, corresponding to the plasmatic levels observed *in vivo* (Physiol), and the levels routinely used in hepatic *in vitro* cultures (Diff) (4) was performed to better understand their effect on hepatocyte biology and phenotype (**Table 1**). HepG2 and hpHeps and undifferentiated cells (hnMSCs) were used as positive and negative controls, respectively.

**Figure 1:**
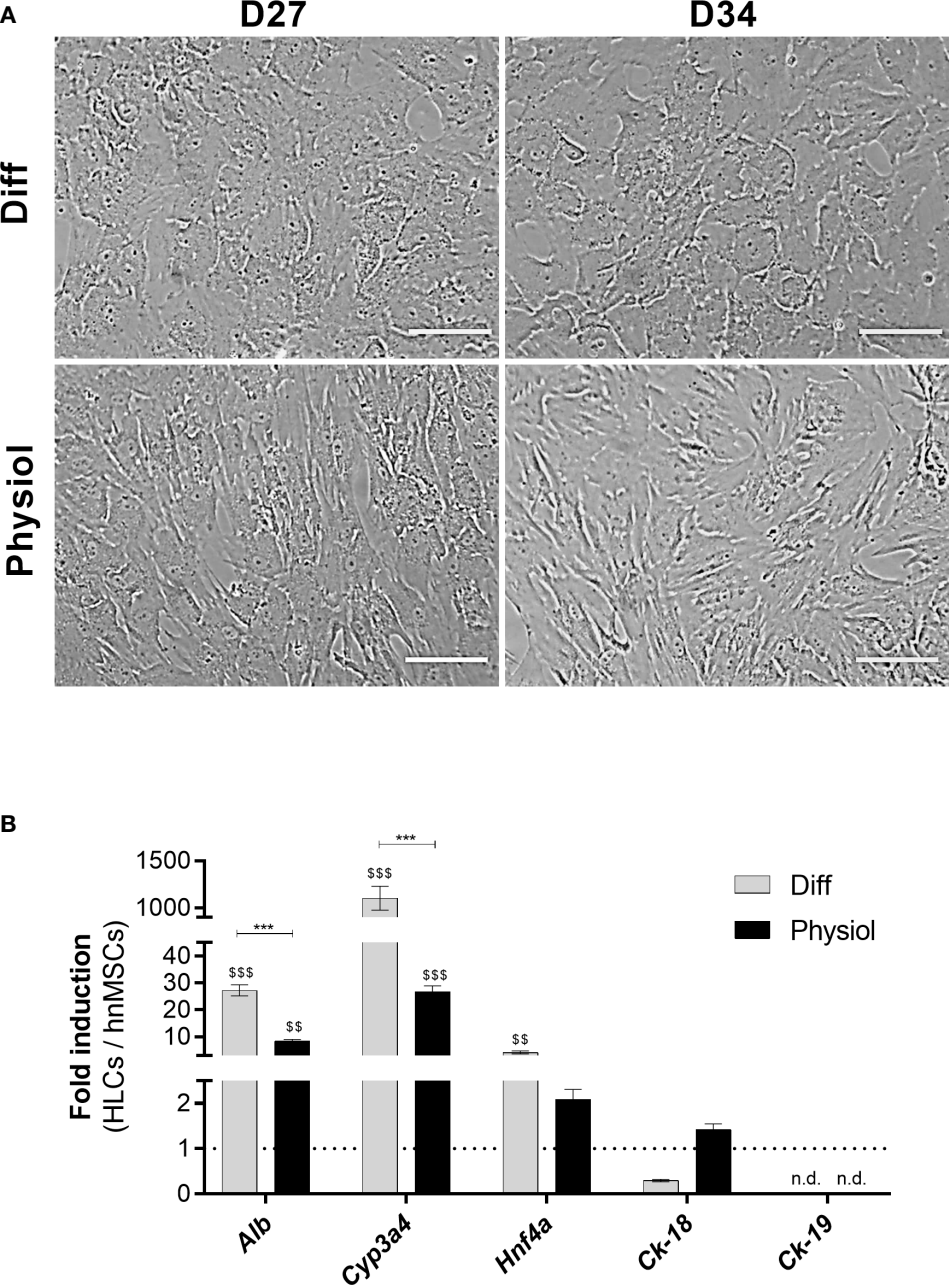
Physiol maintained the typical polygonal hepatocyte-like morphology and the induction of *Alb*, *Cyp3a4* and *Hnf4a* in HLCs. **(A)** Morphology in HLCs maintained in Diff and Physiol, at D27 and D34. Scale bar = 100 µm. **(B)** Hepatic-specific gene expression in HLCs at D34. Data are expressed as fold induction relative to hnMSCs and represented as Average ± SEM (*n* = 3-6). *** significantly differs from the other conditions with *p* < 0.001. $$ and $$$ significantly induced with *p* < 0.01 and *p* < 0.001, respectively (two-way ANOVA). D27, D34, day 27, day 34 of the differentiation protocol; HLCs, hepatocyte-like cells; hnMSCs, undifferentiated human neonatal mesenchymal stem cells; *Alb,* albumin; *Cyp3a4,* cytochrome P450, 3A4; *Hnf4a,* hepatocyte nuclear factor-4α; *Ck-18,* cytokeratin-18; *Ck-19,* cytokeratin-19; n.d., non-determined.

**Figure 2:**
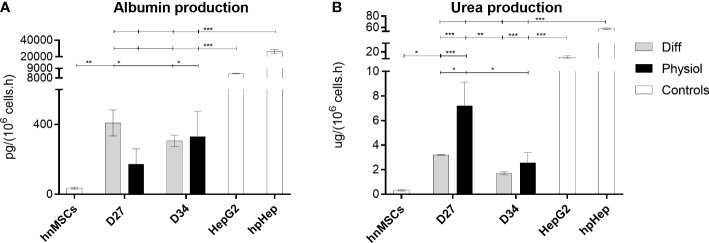
Albumin production was similar in both conditions while urea synthesis was improved with Physiol. Effect of Diff and Physiol and culture time on **(A)** albumin and **(B)** urea production. Data are represented as Average ± SEM (*n* = 3). Undifferentiated hnMSCs and HepG2 cell line and cryopreserved hpHeps are negative and positive controls, respectively (white bars). *, **, *** significantly differs from the other conditions with *p* < 0.05, *p* < 0.01 and *p* < 0.001, respectively (two-way ANOVA). hnMSC, undifferentiated human neonatal mesenchymal stem cells; hpHep, human primary hepatocytes; D27, D34, day 27, day 34 of the differentiation protocol.

Under Physiol conditions, cells displayed a typical polygonal hepatocyte-like shape with one or more nuclei with prominent nucleoli in both days (**Figure 1A**). The expression levels of the hepatic-specific genes *Alb*, *Cyp3a4 and Hnf4a* in HLCs were higher in Diff (*p* < 0.001, *p* < 0.001 and *p* < 0.01, respectively) and in Physiol (*p <* 0.01, *p* < 0.001 and *p* > 0.05, respectively) at D34 relative to hnMSCs (**Figure 1B**). *Ck-18* expression, on the other hand, was similar to that of hnMSCs, whereas the cholangiocyte marker *Ck-19* was not detected in both media. These observations were further supported by identical albumin production in both conditions (**Figure 2A**) and higher levels of urea synthesis at D27 in HLCs maintained in Physiol when compared to Diff (*p* < 0.05) (**Figure 2B**). Curiously, overall, intermediate concentrations of glucose, dexamethasone and insulin (Diff ^-glu^ and Physiol ^+glu^) did not result in an improvement of the HLC phenotype when compared to Physiol (**Supplementary Figures 1, 2**).

**Figure 3:**
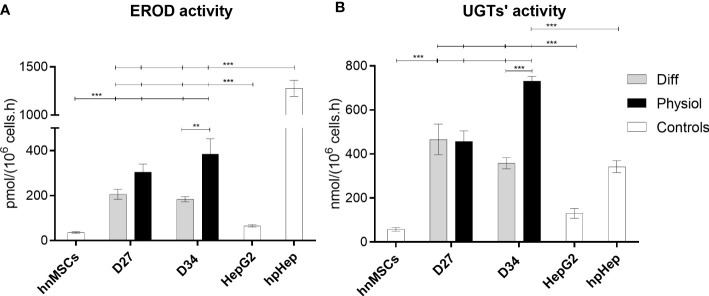
EROD and UGTs’ activities were improved with Physiol. Effect of culture time and medium composition on **(A)** EROD (phase I) and **(B)** UGTs’ (phase II) activities. Data are represented as Average ± SEM (*n* = 3-4). Undifferentiated hnMSCs and HepG2 cell line and cryopreserved hpHep are negative and positive controls, respectively (white bars). **, *** significantly differs from the other conditions with *p* < 0.01 and *p* < 0.001, respectively (two-way ANOVA). EROD, 7-ethoxyresorufin-O-deethylase; UGTs, uridine 5’-diphosphate glucuronosyltransferases; hnMSC, undifferentiated human neonatal mesenchymal stem cells; hpHep, human primary hepatocytes; D27, D34, day 27, day 34 of the differentiation protocol.

**Figure 4:**
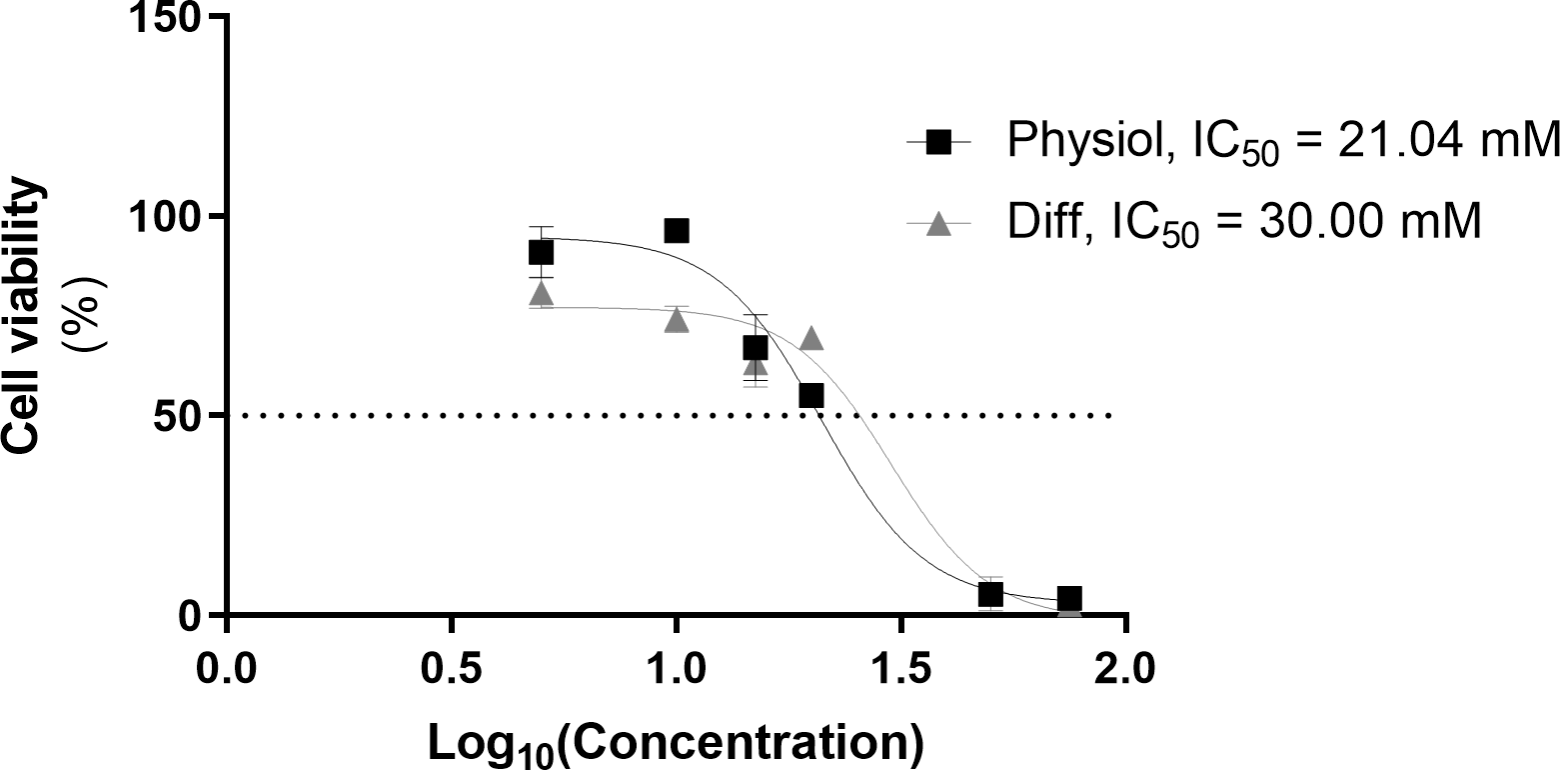
HLCs in Physiol presented higher sensitivity to paracetamol-induced cytotoxicity. Comparison of paracetamol cytotoxicity in HLCs maintained in Diff and Physiol at D34 evaluated by MTS mitochondrial activity assay. HLCs were incubated with 0, 5, 10, 15, 20, 50 and 75 mM of paracetamol. The percentage of viable cells is calculated relative to non-treated HLCs (mean ± SEM) (*n* = 3).

The hepatic phenotype was further assessed by measuring phase I and II enzyme activity. EROD activity, covering CYP1A1 and CYP1A2 activity (12, 18–25), at D34, was higher in HLCs maintained in Physiol than cells in Diff (*p* < 0.01) and HepG2 (*p* < 0.001) (**Figure 3A**). Concerning phase II of biotransformation, UGT activity in HLCs kept in Physiol at D34 was superior to all other conditions, including hpHeps (*p* < 0.001) (**Figure 3B**). These results indicate that HLCs maintained in Physiol were metabolic competent, which was further confirmed by the cells’ ability to metabolize the model drug, paracetamol (**Figure 4**). Cell viability was evaluated by MTS reduction assay and the IC_50_ for HLCs kept in Physiol and in Diff were 21.04 mM and 30.00 mM, respectively, suggesting that HLCs kept in Physiol were more sensitive to paracetamol exposure than in Diff.

### HLCs cultured in a more physiological medium display increased ATP production and maximal mitochondrial functional capacity

**Figure 5:**
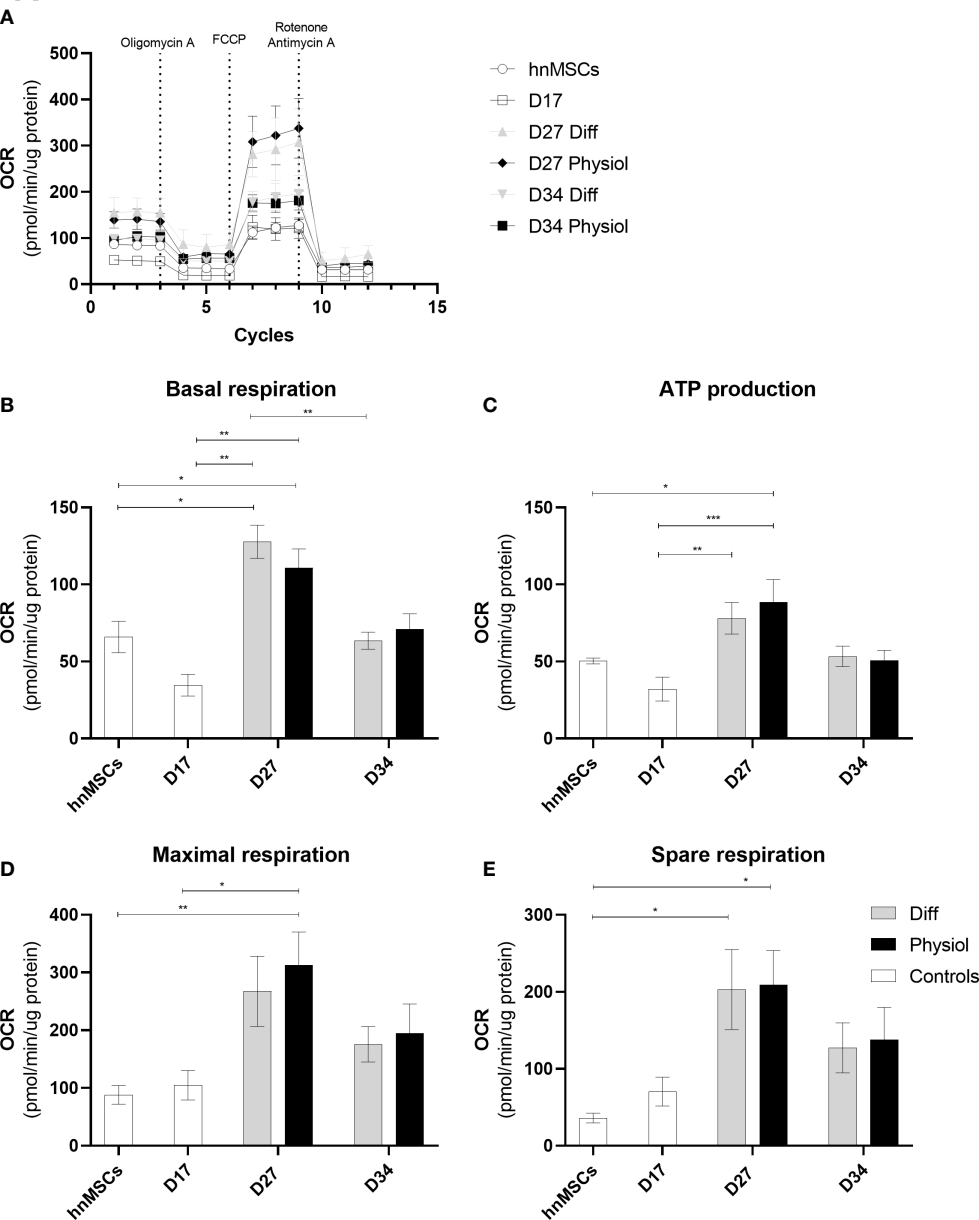
Physiol maintained HLC mitochondrial function. Evaluation of mitochondrial function by directly measuring the OCR of cells at different stages of differentiation and exposed to different media. **(A)** OCR in the presence of 1.5 μM of oligomycin, 1.25 μM of FCCP, 2 μM of antimycin and 2 μM of rotenone. **(B)** Basal respiration. **(C)** ATP production. **(D)** Maximal respiration. **(E)** Spare respiration. Data are represented as Average ± SEM (*n* = 3-5). *, **, *** significantly differs from the other conditions with *p <* 0.05, *p <* 0.01 and *p <* 0.001, respectively (two-way ANOVA). hnMSC, undifferentiated human neonatal mesenchymal stem cells; OCR, oxygen consumption rate; D17, D27, D34, day 17, day 27, day 34 of the differentiation protocol.

To evaluate mitochondrial function in the different cells we used an extracellular flux analyzer (Seahorse XFp). Real time oxygen consumption rates (OCR) in hnMSCs and HLCs were measured using Seahorse XFp Cell Mito Stress Test (**Figure 5A**). Basal respiration (**Figure 5B**) shows the cellular energetic demands under baseline conditions. At D27, HLCs in both media displayed higher basal respiration, being significantly higher than in cells at the hepatoblast phase (D17) (*p <* 0.01) or undifferentiated (*p <* 0.05); whereas at D34 there was a decrease in basal respiration in both conditions (Diff, *p <* 0.01). ATP production (**Figure 5C**) refers to the reduction in OCR upon inhibition of ATP synthase activity. HLCs in Physiol at D27 had a significantly higher ATP production than cells at D17 and hnMSCs (*p <* 0.001 and *p <* 0.05, respectively). Maximal respiration (**Figure 5D**), on the other hand, demonstrates the maximum rate of respiration that the cell can achieve by stimulating the respiratory chain to operate at maximum capacity and it was significantly higher in HLCs in Physiol at D27 when compared to hnMSCs (*p <* 0.01) and cells at D17 (*p <* 0.05). Finally, spare respiration (**Figure 5E**) indicates cell fitness or flexibility to respond to an energetic demand as well as how closely the cell is to respire at its maximum capacity. Herein, HLCs in both Physiol and Diff at D27 displayed enhanced cell fitness when compared to undifferentiated cells (*p <* 0.05). Importantly, neither of the intermediate conditions tested presented improved mitochondrial activity when compared to Physiol (**Supplementary Figure 4**). These data support the higher differentiation degree of HLCs in Physiol throughout time, which is known to be associated with higher rates of mitochondria respiration when compared to undifferentiated cells.

### HLCs under more physiological conditions display insulin-responsive glucose metabolism

**Figure 6:**
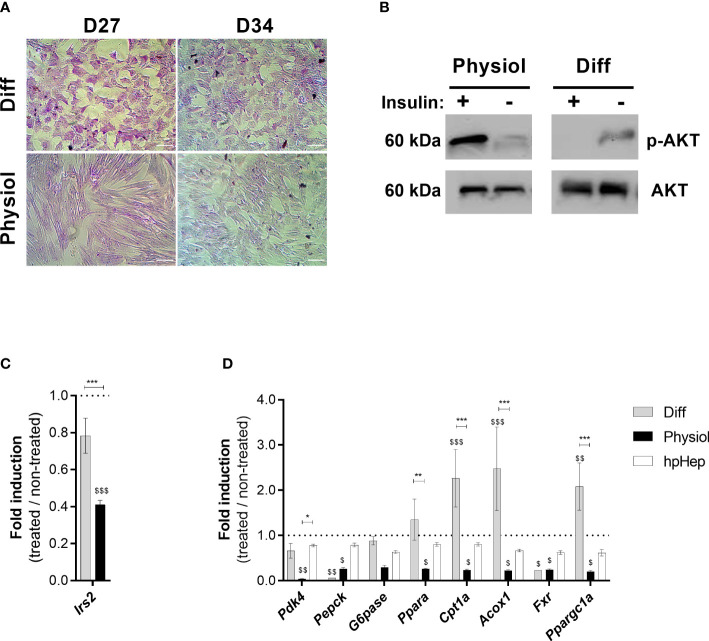
Physiol maintained glycogen storage ability and improved HLC responsiveness to insulin stimuli. **(A)** Glycogen storage ability evaluated by PAS staining at D27 and D34. Scale bar = 100 µm. **(B)** AKT phosphorylation confirmed in HLCs kept in Physiol in response to insulin by Western Blot analysis. **(C)** Effect of insulin in *Irs2* expression in HLCs subjected to fasting, evaluated by RT-qPCR. **(D)** Effect of insulin in HLC gene expression. Genes involved in glycolysis (*Pdk4*), gluconeogenesis (*Pepck* and *G6pase*), fatty acid oxidation (*Ppara*, *Cpt1a* and *Acox1*), bile acid metabolism (*Fxr*) and mitochondrial function (*Ppargc1a*) were evaluated by RT-qPCR. RT- qPCR data are expressed as fold induction relative to non-treated cells (*n* = 3-6). *, **, *** significantly differs from the other conditions with *p <* 0.05, *p <* 0.01 and *p <* 0.001, respectively. $, $$ and $$$ significantly induced or repressed with *p <* 0.05, *p <* 0.01 and *p <* 0.001, respectively (two-way ANOVA). hpHep, human cryopreserved hepatocytes; *Pdk4*, pyruvate dehydrogenase kinase 4; *Pepck* phosphoenolpyruvate carboxylase; *G6pase*, glucose-6-phosphatase; *Ppara*, peroxisome proliferator-activated receptor α; *Cpt1a*, carnitine palmitoyltransferase 1α; *Acox1*, acyl-CoA oxidase 1; *Fxr*, Farnesoid X receptor; *Ppargc1a*, peroxisome proliferator-activated receptor γ coactivator 1-α; *Irs2*, insulin receptor substrate 2.

To evaluate HLC insulin signaling we measured glycogen storage ability (**Figure 6A**), AKT phosphorylation (**Figure 6B**) and insulin receptor substrate 2 (*Irs2*) expression (**Figure 6C**). Moreover, the effects of insulin stimuli on the expression of genes involved in glycolysis (*Pdk4*), gluconeogenesis (*Pepck* and *G6pase*), FA oxidation (*Ppara*, *Cpt1a* and *Acox1*), bile acid metabolism (*Fxr*) and mitochondrial function (*Ppargc1a*) was also evaluated (**Figure 6D**).

All conditions displayed glycogen storage capacity (**Figure 6A** and **Supplementary Figure 5**). Most importantly, we could only observe insulin-stimulated AKT phosphorylation in HLCs kept in Physiol (**Figure 6B**). Moreover, upon insulin exposure, *Irs2* expression was downregulated in HLCs in Physiol (**Figure 6C**, *p* < 0.001). Likewise, HLCs in Physiol exposed to insulin presented a downregulation of *Pdk4*, *G6pase*, *Ppara*, *Cpt1a*, *Acox1*, *Fxr* and *Ppargc1a*, indicating an induction of glycolysis and bile acid metabolism (*Pdk4* and *Fxr* are negative regulators of these pathways, respectively) and inhibition of gluconeogenesis and FA oxidation (**Figure 6D**). *Ppargc1a* enhances the expression of genes related to mitochondrial function and induces gluconeogenesis and, herein, its downregulation also reinforces gluconeogenesis inhibition. On the other hand, upon insulin incubation, HLCs in Diff maintained the expression of *Pdk4* and *G6pase* and increased the expression of *Cpt1a*, *Acox1* and *Ppargc1a* which is associated to FA oxidation and mitochondrial function induction. Overall, our data suggest that HLCs in Physiol were more sensitive to insulin stimulation than hpHep.

## Discussion

Stem-cell based hepatic *in vitro* models have demonstrated potential for studying and modeling metabolic-related diseases (30, 31). However, most hepatic differentiation protocols maintain differentiated HLCs in micromolar concentrations of insulin and dexamethasone and millimolar concentrations of glucose that reduce insulin sensitivity. Thus, this work focused on the modulation of insulin signaling and the metabolism of glucose and the synthetic glucocorticoid dexamethasone towards the development of a human-based hepatic *in vitro* model suitable for studying insulin signaling pathway.

As such, prior to the evaluation of the insulin-regulated metabolism, the MSC-derived HLC basal hepatic phenotype and functionality in more physiological concentrations of insulin, glucose and dexamethasone was compared to Diff (4), and the typical hepatic polygonal shape morphology, overexpression of hepatic-specific genes, albumin production and mitochondrial function confirmed.

As for hepatic specific genes, the expression of the hepatic marker, *Ck-18*, was detected in HLCs and in hnMSCs, possibly due to the presence of early endodermal markers in umbilical cord matrix-derived MSCs populations (32); whereas the cholangiocyte marker *Ck-19* was not expressed in HLCs in Physiol confirming the hepatocyte lineage commitment. Additionally, overexpression of *Alb* or *Hnf4a* in HLCs showed that the concentrations of glucose, insulin and dexamethasone herein used maintained the HLC differentiated status without interfering with HLC viability and adherence, as previously demonstrated for human primary hepatocytes (8). Likewise, albumin production was similar in HLCs in both conditions, while Physiol could improve ammonia detoxification as demonstrated by urea production. It was previously reported that lower glucose concentrations result in higher urea production in HepG2-C3A (33) and in this work, we could also observe that media with lower glucose concentrations (Physiol and Diff ^-glu^) demonstrated the same trend (**Supplementary Figure 2B**). Moreover, glucagon was shown to increase urea production in hpHeps (34). Since glucagon and insulin are antagonistic hormones, media with lower insulin concentrations may have a positive impact in HLC ammonia detoxification.

As for the biotransformation ability, *Cyp3a4* expression level of HLCs was increased in all Diff conditions when compared to Physiol (Physiol and Physiol ^+glu^), which may be a consequence of the higher concentrations of dexamethasone in Diff media, a known CYP3A4 inducer (**Supplementary Figure 1B**) (35). In addition, phase I and phase II activities were evaluated through EROD (CYP1A1/2) and UGT assays, respectively. In Physiol, both activities were enhanced, further supporting the improvement of HLC hepatic features under these conditions. This is in accordance with previous observations showing that dexamethasone decreases CYP1A1 and CYP1A2 activity in hpHeps (12). Herein, glucose may also have negatively regulated EROD activity, given that at D34, EROD activity in HLCs kept in low glucose levels (Physiol and Diff ^-glu^) was higher than HLCs in Physiol ^+glu^ and Diff (media with 25 mM of glucose), as seen in **Figure 3A** and **Supplementary Figure 3A**. Indeed, Davidson et al. reported that hypoglycemic conditions (~0.4 – 0.5 mM of glucose) significantly increased the expression of *Cyp1a2*, as compared to a normoglycemic control (~5 mM) (14). Interestingly, in our protocol, cells maintained in lower glucose concentrations also showed higher CYP1A1/2 activities.

To further validate the usefulness of our model for hepatotoxicity studies, HLCs were exposed to paracetamol, a drug known to cause hepatotoxicity in overdose (36–38) and whose toxicity is dependent on phase I and II metabolism. At high-dose paracetamol exposure, the metabolic pathway switches from the detoxifying phase II metabolism to phase I metabolism, generating the toxic intermediate *N*-acetyl-*p*-benzoquinone imine (NAPQI). Paracetamol is mainly metabolized through conjugation with sulphate and glucuronic acid and, to a lesser extent, through oxidation by CY2E1, CYP1A2 and CYP3A4 (36). Accordingly, cell viability upon paracetamol exposure was assessed as an indirect measure of HLC metabolic competence. Indeed, the higher paracetamol cytotoxicity observed in Physiol is an indicator of higher biotransformation ability. Our results demonstrate that decreased concentrations of insulin, glucose and dexamethasone improved the sensitivity of HLCs to paracetamol toxicity, suggesting a higher phase I activity, in accordance with EROD activity results, and consequently higher formation of the toxic metabolite NAPQI. Moreover, changes in the metabolic cell status can result in different drug efficacy and safety. Therefore, the observed differences between Diff and Physiol in drug metabolism suggest that our HLC model could be able to recapitulate differences in CYP450 activities observed in numerous metabolic pathologies, namely NAFLD (39, 40).

Insulin regulates glucose and lipid metabolism in hepatocytes and mitochondria are the central hub for controlling metabolic homeostasis and energy production. Disturbances in mitochondrial function mediate hepatocyte injury, affect cell viability, and are associated with NAFLD, drug-induced hepatotoxicity, and cholestasis (37, 41). Herein, Physiol maintained HLC mitochondrial functionality under basal conditions, as well as the mitochondrial contribution for ATP production, flexibility to respond to energetic demands and the maximum operation capacity when compared to Diff. These results can also confirm that Physiol could maintain the hepatic differentiation degree as it was reported that glycolysis is the main provider of ATP in stem cells while mitochondrial oxidative phosphorylation is the most predominant source of ATP in differentiated cells (42).

Finally, carbohydrate metabolism and insulin signaling were specifically assessed in HLCs as the liver regulates blood glucose levels through glycogen synthesis and gluconeogenesis. Upon insulin binding to the insulin receptor, there is a cascade of phosphorylation of downstream enzymes: insulin receptor substrate proteins, PI3K and AKT. AKT phosphorylation is an important event in the insulin transduction pathway as it will then mediate the effects of insulin on glucose, glycogen, protein and lipid metabolism (43). Herein, HLCs were indeed able to form glycogen stores in both conditions. Moreover, in response to insulin exposure, only HLCs in Physiol exhibited AKT phosphorylation. The absence of AKT phosphorylation in HLCs in Diff, a signal of insulin resistance development, may be related to the chronic exposure to high insulin concentrations (1.72 μM), as previously reported (44, 45). IRS2 plays a major role in hepatic energy homeostasis in fasting conditions, mediating insulin effects through the AKT cascade. *Irs2* expression increases in the fasting state and immediately decreases after food intake which is accompanied by an increase in insulin blood levels (46). Accordingly, after subjecting HLCs to fasting and subsequent exposure to insulin, only HLCs in Physiol presented a downregulation of *Irs2* as previously described (46–48). In particular, under non-pathological conditions, insulin will induce glycolysis and lipid synthesis while inhibiting gluconeogenesis and FA oxidation (49). Accordingly, a RT-qPCR analysis showed that HLCs, maintained under lower concentrations of insulin, dexamethasone and glucose throughout the maturation phase (Physiol), were more sensitive to insulin stimuli, upregulating glycolysis and bile acid metabolism and downregulating gluconeogenesis, FA oxidation and mitochondrial function-related pathways as observed *in vivo*. On the other hand, HLCs maintained in higher and non-physiological concentrations of insulin, dexamethasone and glucose (Diff) were not responsive to insulin insult, keeping the expression of genes involved in glycolysis and gluconeogenesis while FA oxidation is induced. Therefore, lower concentrations of those supplements are best suited for maintaining cells for energy metabolism regulation studies by maintaining insulin responsiveness.

Importantly, the activation of glycolysis and bile acid synthesis along with the inhibition of gluconeogenesis and FA oxidation accompanied by increased biotransformation activity are zonation features characteristic of perivenous hepatocytes (50). Therefore, overall, these results may suggest the modulation of HLCs towards a perivenous-like phenotype, important when studying metabolic diseases. NAFLD, in particular, has a perivenous predominance due to reduced FA oxidation gene expression that will lead to faster lipid accumulation in this region (51). In particular, we observe that our HLC model displays glycogen storage capacity and modulates gluconeogenic gene expression, which was a limitation found by Nagarajan and colleagues in different hepatocyte lines (1).

Moreover, in contrast, to other works that use FBS, insulin at micromolar concentrations, glucose concentrations above 10 mM and dexamethasone concentrations superior to 100 nM, which may affect insulin action (30, 31, 52–54), the model herein developed represents a more physiological system that can be used in the future to induce NAFLD and study disease mechanisms. Indeed, to our knowledge, it is shown for the first time the AKT phosphorylation, downregulation of *Irs2* and of genes related to glucose and lipid metabolism upon insulin exposure in a HLCs *in vitro* model.

In sum, our data reveal that more physiological levels of insulin, glucose and dexamethasone regulate insulin signaling and energy metabolism in HLCs as it improves insulin sensitivity along with ammonia detoxification, biotransformation activity and mitochondrial function while retaining albumin production and overexpression of hepatic-specific genes. Therefore, manipulating glucose homeostasis and glucocorticoid concentration can be a strategy for developing relevant hepatic *in vitro* models for drug metabolism studies, hepatotoxicity assessment and disease modeling of energy metabolism-related disorders.

## Data availability statement

The original contributions presented in the study are included in the article/**Supplementary Material**. Further inquiries can be directed to the corresponding author.

## Author contributions

JM, JRu and VM developed the study concept and the study design. JRo, AF-P, SC and AS performed the experiments and data collection. JRo and AF-P performed the data analysis and interpretation under the supervision of JM and VM. JM and JRo drafted the manuscript. JRu and VM provided critical revisions. All authors contributed to the article and approved the submitted version.
