# Supplementary material for: Improving human mesenchymal stem cell-derived hepatic cell energy metabolism by manipulating glucose homeostasis and glucocorticoid signaling
Source: Front Endocrinol (Lausanne). 2023 Jan 13;13:1043543. doi: 10.3389/fendo.2022.1043543 (PMC9880320; doi:10.3389/fendo.2022.1043543)
Supplement: Supplementary file 1 [file DataSheet_1.docx]

Supplementary Material

# Supplementary Figures


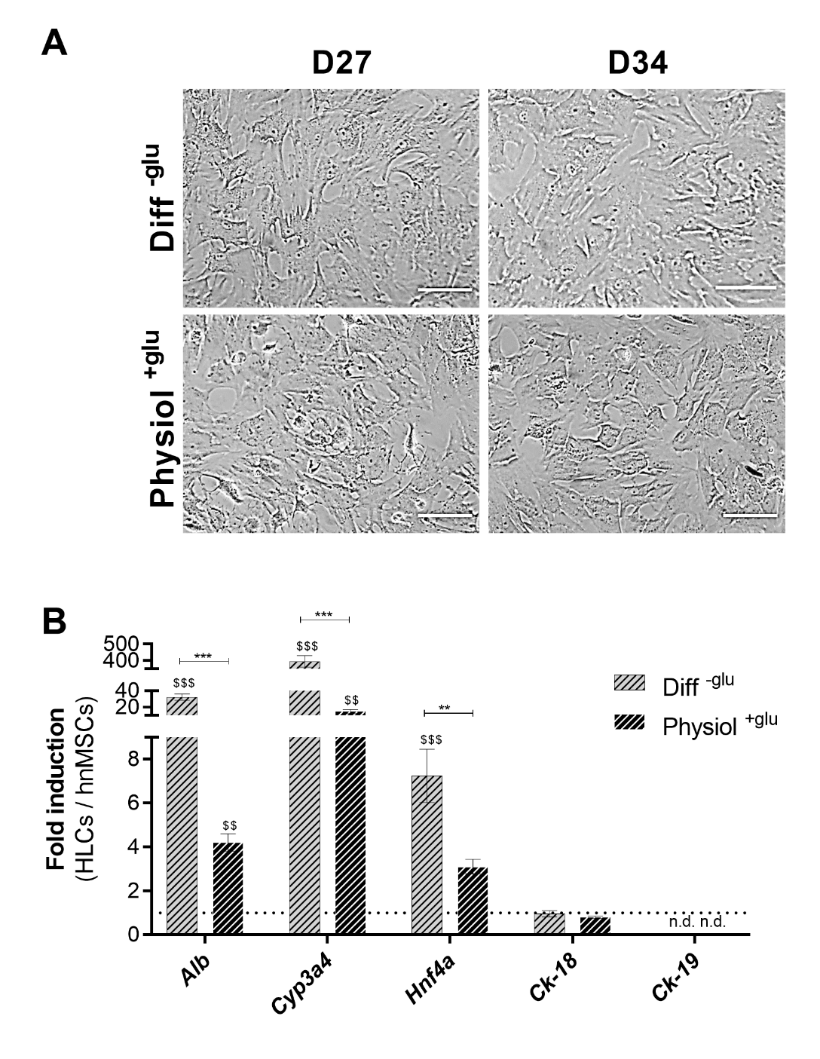


**Supplementary Figure 1.** (A) HLC morphology (scale bar = 100 µm) and (B) expression of hepatic-specific genes. Ck-19 expression was undetermined in both conditions. Data are normalized to the reference gene β-actin, expressed as fold induction relative to hnMSCs are represented as Average ± SEM (n = 3-6). Grid line represents fold induction equal to 1. **, *** significantly differs from the other conditions with *p* < 0.01 and *p* < 0.001, respectively. $$ and $$$ significantly induced with *p* < 0.01 and *p* < 0.001, respectively (two-way ANOVA). Abbreviations: D27, D34 (day 27, day 34 of the differentiation protocol); HLCs (hepatocyte-like cells); hnMSCs (undifferentiated human neonatal mesenchymal stem cells); Diff^-glu^ (Diff containing 5 mM glucose); Physiol^+glu^ (Physiol containing 25 mM glucose); *Alb* (albumin); *Cyp3a4* (cytochrome P450 3A4); *Hnf4a* (hepatocyte nuclear factor-4α); *Ck-18* (cytokeratin-18); *Ck-19* (cytokeratin-19); n.d. (non-determined).


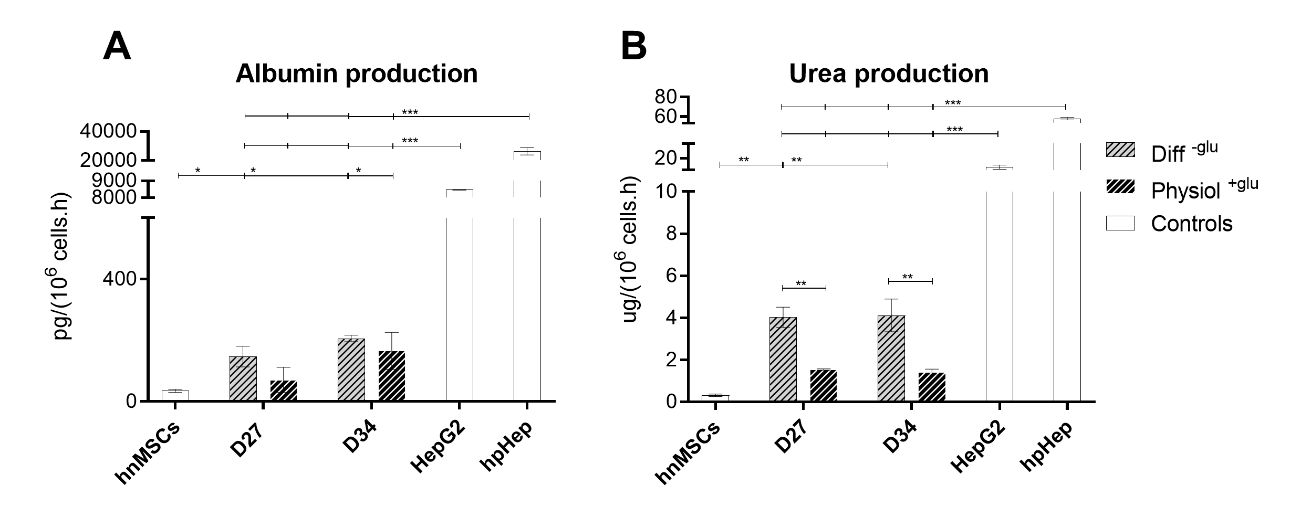


**Supplementary Figure 2.** (A) albumin and (B) urea production. Data are represented as Average ± SEM (n = 3). Undifferentiated hnMSCs and HepG2 cell line and cryopreserved hpHeps are negative and positive controls, respectively (white bars). *, **, *** significantly differs from the other conditions with *p* < 0.05, *p* < 0.01 and *p* < 0.001, respectively (two-way ANOVA). Abbreviations: hnMSC (undifferentiated human neonatal mesenchymal stem cells); hpHep (human primary hepatocytes); Diff^-glu^ (Diff containing 5 mM glucose); Physiol^+glu^ (Physiol containing 25 mM glucose); D27, D34 (day 27, day 34 of the differentiation protocol).


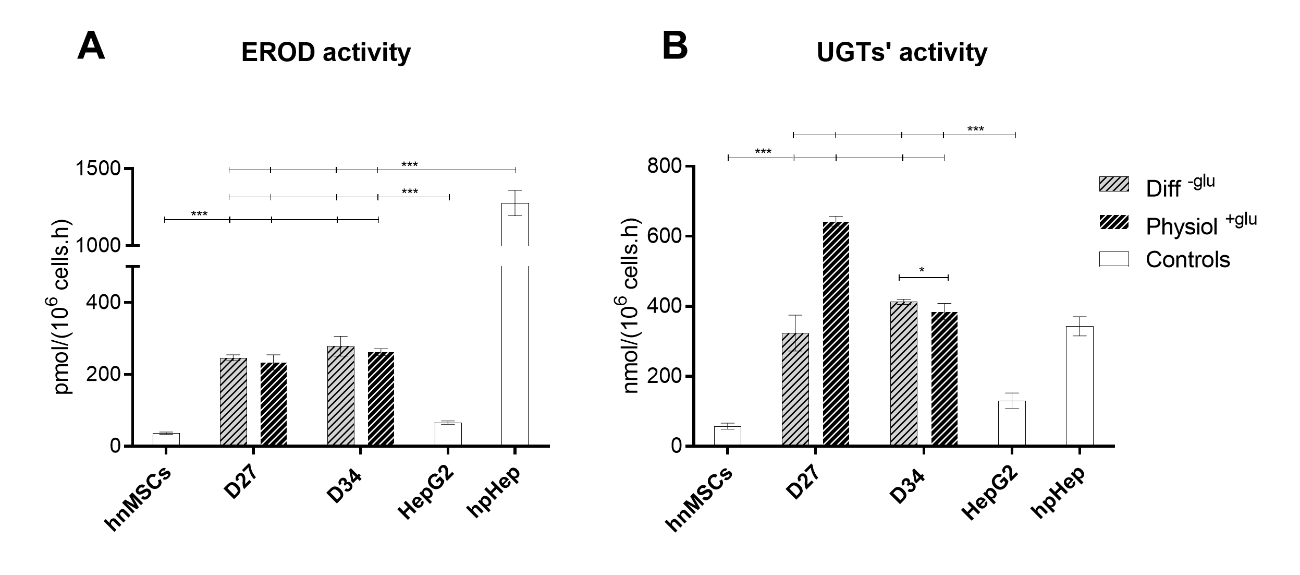


**Supplementary Figure 3.** Biotransformation activity in intermediate conditions. Data are represented as Average ± SEM (n = 3-4). Undifferentiated hnMSCs and HepG2 cell line and cryopreserved hpHep are negative and positive controls, respectively (white bars). *, *** significantly differs from the other conditions with *p* < 0.05 and *p* < 0.001, respectively (two-way ANOVA). Abbreviations: EROD (7-ethoxyresorufin-O-deethylase); UGTs (uridine 5’-diphosphate glucuronosyltransferases); hnMSC (undifferentiated human neonatal mesenchymal stem cells); hpHep (human primary hepatocytes); Diff^-glu^ (Diff containing 5 mM glucose); Physiol^+glu^ (Physiol containing 25 mM glucose); D27, D34 (day 27, day 34 of the differentiation protocol).


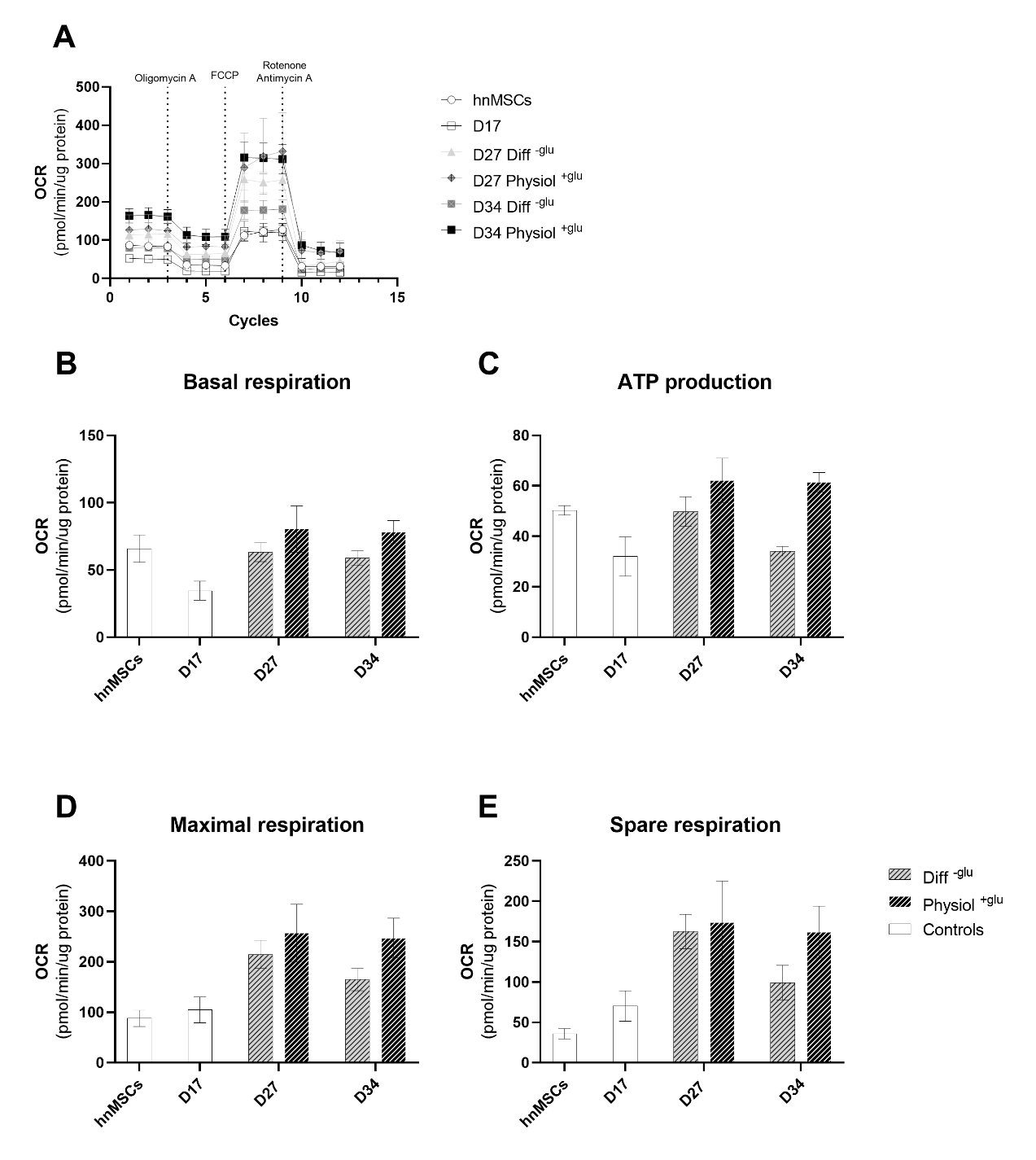


**Supplementary Figure 4.** Mitochondrial function in intermediate conditions. OCR in the presence of 1.5 µM of oligomycin, 1.25 µM of FCCP, 2 µM of antimycin and 2 µM of rotenone in HLCs in different conditions at D27 (A) and at D34 (B). (C) Basal respiration. (D) ATP production. (E) Maximal respiration. (F) Spare respiration. Data are represented as Average ± SEM (n = 3-5). Abbreviations: hnMSC (undifferentiated human neonatal mesenchymal stem cells); OCR (oxygen consumption rate); Diff^-glu^ (Diff containing 5 mM glucose); Physiol^+glu^ (Physiol containing 25 mM glucose); D17, D27, D34 (day 17, day 27, day 34 of the differentiation protocol).


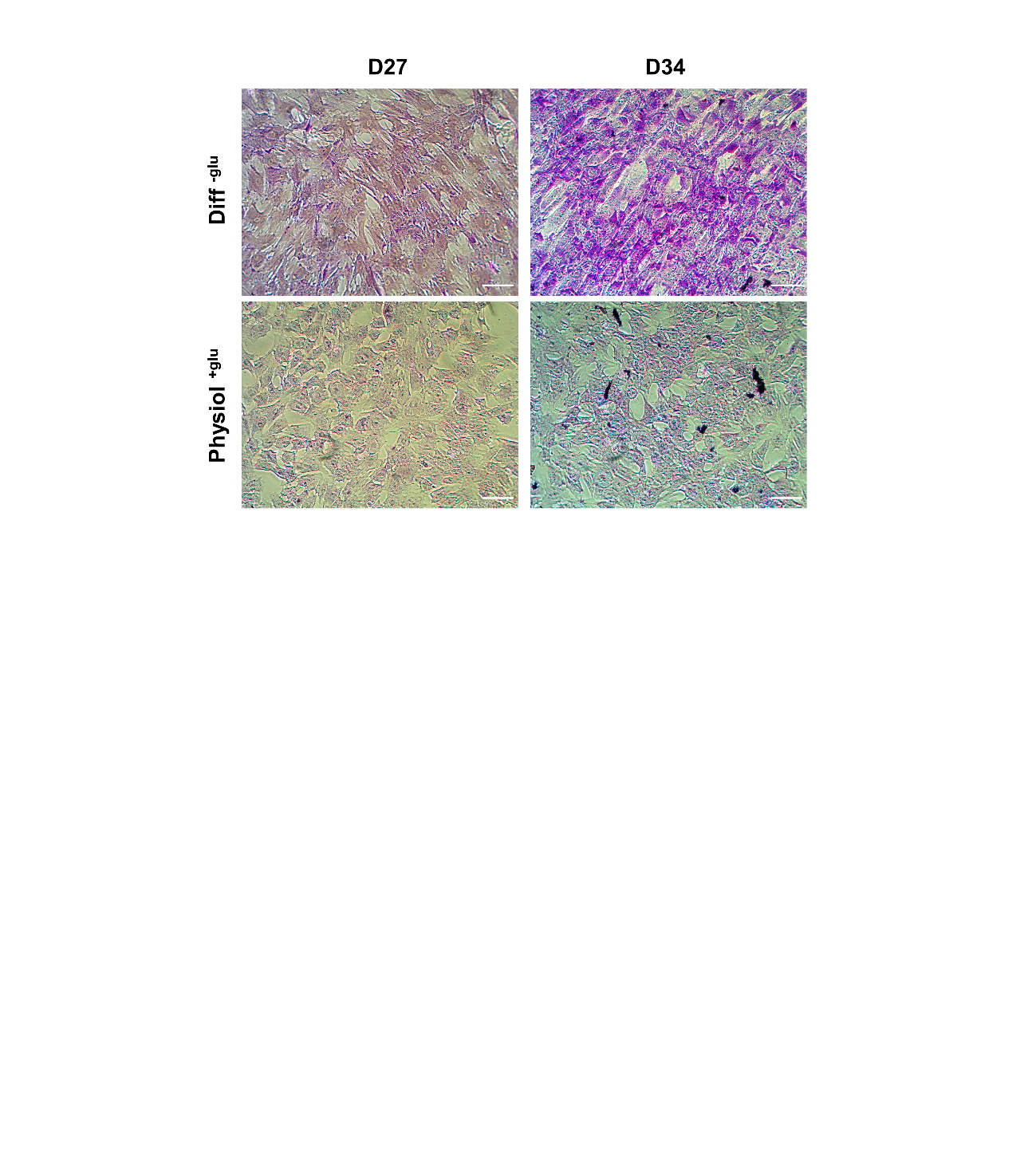


**Supplementary Figure 5.** Glycogen storage ability. Scale bar = 100 µm. Abbreviations: Diff^-glu^ (Diff containing 5 mM glucose); Physiol^+glu^ (Physiol containing 25 mM glucose); D27, D34 (day 27, day 34 of the differentiation protocol).
